# Supplementary material for: Antimicrobial Resistance of Salmonella Strains Isolated from Human, Wild Boar, and Environmental Samples in 2018–2020 in the Northwest of Italy
Source: Pathogens. 2022 Nov 30;11(12):1446. doi: 10.3390/pathogens11121446 (PMC9787983; doi:10.3390/pathogens11121446)
Supplement: Supplementary file 1 [file pathogens-11-01446-s001.zip › pathogens-2043143-supplementary.pdf]

Table S1. Frequency of AMR profiles in *Salmonella* subtypes and serotypes identified in human and wild boar infections and in environmental samples.

Source	*Serotype*	*AMR profile*	*N. isolates*	*%*
Environment	6,7:c:1,5	STR	1	0.4
Environment	6,7:c:1,5	STR-SSS	1	0.4
Environment	Typhimurium 1.4.[5].12:i:-	AMP-STR-SSS-TET	1	0.4
Environment	Typhimurium 1.4.[5].12:i:-	TET-TGC	3	1.2
Environment	Brandenburg	SSS-TET	1	0.4
Environment	Cerro	SSS	1	0.4
Environment	Coeln	SSS	1	0.4
Environment	Coeln	SSS-TET	1	0.4
Environment	Eingedi	SSS-TET	1	0.4
Environment	Fann	SSS	1	0.4
Environment	Goldcoast	AZI-CIP-TET-TGC-SXT	1	0.4
Environment	Ilugun	STR	1	0.4
Environment	Kapemba,	GEN	1	0.4
Environment	Kottbus,	SSS	2	0.8
Environment	London	AZI-GEN-SXT	1	0.4
Environment	Napoli	SSS	4	1.5
Environment	Napoli	SSS-SXT	2	0.8
Environment	Rissen	AZI-GEN-TET-TGC-SXT	1	0.4
Environment	Stourbridge	AMC-AMP	1	0.4
Environment	Stourbridge	AZI-GEN-SXT	2	0.8
Environment	Stourbridge	SSS	1	0.4
Environment	Thompson	SSS	2	0.8
Environment	Thompson	STR	1	0.4
Environment	Thompson	STR-SSS	1	0.4
Environment	Veneziana	AMC-GEN	1	0.4
Environment	Veneziana	AZI-GEN-SXT	1	0.4
Environment	Veneziana	GEN	1	0.4
Environment	Veneziana	SSS	7	2.7
Environment	S. enterica subsp. enterica (non-typed)	GEN-SSS	1	0.4
Environment	S. enterica subsp. enterica (non-typed)	SSS	5	1.9
Environment	S. enterica subsp. enterica (non-typed)	STR	1	0.4
Environment	S. enterica subsp. enterica (non-typed)	STR-SSS	2	0.8
Environment	S. enterica subsp. enterica (non-typed)	AZI-CIP-GEN-SXT	1	0.4
Environment	S. enterica subsp. enterica (non-typed)	AZI-GEN-SXT	1	0.4
Environment	S. enterica subsp. enterica (non-typed)	GEN-SSS-TET	1	0.4
Environment	S. enterica subsp. enterica (non-typed)	GEN-SSS-SXT	1	0.4
Environment	S. enterica subsp. enterica (non-typed)	GEN-STR-SSS	1	0.4
Environment	S. enterica subsp. enterica (non-typed)	SSS	5	1.9
Environment	S. enterica subsp. enterica (non-typed)	SSS-SXT	3	1.2
Environment	S. enterica subsp. salamae	SSS	1	0.4
Environment	S. enterica subsp. salamae	STR-SSS	1	0.4
Environment	S. enterica subsp. diarizonae	STR	5	1.9
Human	6,7:c:1,5	AMP-CHL-STR	1	0.4
Human	6,7:c:1,5	AMP-CHL-STR-SSS-TET-SXT	1	0.4
Human	6,7:c:1,5	AMP-STR-SSS-NAL-SXT	1	0.4
Human	Typhimurium 1.4.[5].12:i:-	AMC-AMP-CHL-STR-SSS-TET	1	0.4

Human	Typhimurium 1.4.[5].12:i:-	AMC-AMP-CHL-STR-TET-TGC	2	0.8
Human	Typhimurium 1.4.[5].12:i:-	AMC-AMP-FOX-CHL-STR-SSS-TET	1	0.4
Human	Typhimurium 1.4.[5].12:i:-	AMC-AMP-STR-TET-TGC	3	1.2
Human	Typhimurium 1.4.[5].12:i:-	AMC-STR-TET-TGC	1	0.4
Human	Typhimurium 1.4.[5].12:i:-	AMP-CHL-STR-SSS-TET	8	3.1
Human	Typhimurium 1.4.[5].12:i:-	AMP-CHL-STR-SSS-SXT	2	0.8
Human	Typhimurium 1.4.[5].12:i:-	AMP-CHL-STR-TET-TGC	4	1.5
Human	Typhimurium 1.4.[5].12:i:-	AMP-FOX-TAZ-STR-SSS	1	0.4
Human	Typhimurium 1.4.[5].12:i:-	AMP-FOX-TAZ-STR-SSS-TET	1	0.4
Human	Typhimurium 1.4.[5].12:i:-	AMP-GEN-STR-TET-TGC	3	1.2
Human	Typhimurium 1.4.[5].12:i:-	AMP-SSS	3	1.2
Human	Typhimurium 1.4.[5].12:i:-	AMP-SSS-TET	1	0.4
Human	Typhimurium 1.4.[5].12:i:-	AMP-STR	1	0.4
Human	Typhimurium 1.4.[5].12:i:-	AMP-STR-SSS	3	1.2
Human	Typhimurium 1.4.[5].12:i:-	AMP-STR-SSS-TET	13	5.0
Human	Typhimurium 1.4.[5].12:i:-	AMP-STR-SSS-SXT-	5	1.9
Human	Typhimurium 1.4.[5].12:i:-	AMP-STR-TET	1	0.4
Human	Typhimurium 1.4.[5].12:i:-	AMP-STR-TET-TGC	14	5.4
Human	Typhimurium 1.4.[5].12:i:-	AMP-TAZ-STR-SSS-TET	1	0.4
Human	Typhimurium 1.4.[5].12:i:-	AMP-TAZ-STR-TET	1	0.4
Human	Typhimurium 1.4.[5].12:i:-	AMP-TET	9	3.5
Human	Typhimurium 1.4.[5].12:i:-	CHL-TET-TGC	1	0.4
Human	Typhimurium 1.4.[5].12:i:-	SSS	6	2.3
Human	Typhimurium 1.4.[5].12:i:-	SSS-NAL	1	0.4
Human	Typhimurium 1.4.[5].12:i:-	SSS-TET	3	1.2
Human	Typhimurium 1.4.[5].12:i:-	STR	1	0.4
Human	Typhimurium 1.4.[5].12:i:-	STR-TET-TGC	2	0.8
Human	Typhimurium 1.4.[5].12:i:-	TET	1	0.4
Human	Typhimurium 1.4.[5].12:i:-	TET-TGC	6	2.3
Human	Brandenburg	SSS	1	0.4
Human	Bredeney	AMC-STR-SSS-TET	1	0.4
Human	Bredeney	SSS	1	0.4
Human	Coeln	AMP-SSS	1	0.4
Human	Coeln	STR	1	0.4
Human	Coeln	TAZ-SSS	1	0.4
Human	Derby	SSS	1	0.4
Human	Derby	SSS-TET	1	0.4
Human	Derby	TET-TGC	1	0.4
Human	Drogana	SSS	1	0.4
Human	Enteritidis	AMP-STR-SSS-NAL	1	0.4
Human	Enteritidis	SSS	2	0.8
Human	Enteritidis	SSS-NAL	1	0.4
Human	Give	SSS-SXT	1	0.4
Human	Goldcoast	AMP-CHL-STR-SSS	1	0.4
Human	Hadar	AMP-STR-SSS-TET-NAL	1	0.4
Human	Infantis	AMP-SSS-TET-NAL	1	0.4
Human	Infantis	CIP	1	0.4
Human	Infantis	CIP-STR-SSS-TET-NAL-SXT	2	0.8

Human	Infantis	SSS	1	0.4
Human	Infantis	SSS-TET-NAL	1	0.4
Human	Kentucky	AMP-CIP-GEN-STR-SSS-TET-NAL	1	0.4
Human	Mbandaka	GEN	1	0.4
Human	Napoli	AMP-STR-SSS	1	0.4
Human	Napoli	AMP-TAZ-CHL-STR-SSS-TET	1	0.4
Human	Napoli	GEN	2	0.8
Human	Napoli	SSS	5	1.9
Human	Othmarschen	AMP-FOX-TAZ-CIP-SSS	1	0.4
Human	Othmarschen	SSS	1	0.4
Human	Rissen	AMP-GEN-TET-TGC	2	0.8
Human	Rissen	AMP-CIP-TET-NAL	1	0.4
Human	Rissen	CHL-TET-TGC	1	0.4
Human	Rissen	SSS-TET	2	0.8
Human	Rissen	STR-SSS	1	0.4
Human	Rissen	TET	2	0.8
Human	Rissen	TET-TGC	1	0.4
Human	Senftenberg	FOT	1	0.4
Human	Thompson	SSS	1	0.4
Human	Thompson	TAZ	1	0.4
Human	Typhi	AMP-CHL-CIP-STR-SSS-NAL-SXT	1	0.4
Human	Typhi	CIP-NAL	1	0.4
Human	Typhimurium	AMC-AMP-CHL-STR-SSS-SXT	1	0.4
Human	Typhimurium	AMC-AMP-CHL-STR-TET-TGC	2	0.8
Human	Typhimurium	AMC-AMP-CIP-CHL-GEN-STR-TET-TGC	1	0.4
Human	Typhimurium	AMC-AMP-STR-SSS-TET-NAL	1	0.4
Human	Typhimurium	AMP	1	0.4
Human	Typhimurium	AMP-CHL	1	0.4
Human	Typhimurium	AMP-CHL-SSS-TET	1	0.4
Human	Typhimurium	AMP-CHL-STR-SSS-TET	5	1.9
Human	Typhimurium	AMP-SSS	1	0.4
Human	Typhimurium	AMP-STR-SSS-TET	1	0.4
Human	Typhimurium	AMP-TAZ-CIP-CHL-STR-SSS-TET-NAL	1	0.4
Human	Typhimurium	FOX-SSS	1	0.4
Human	Typhimurium	SSS	2	0.8
Human	Typhimurium	STR-SSS-TET	2	0.8
Human	Typhimurium	STR-TET-TGC	2	0.8
Human	Virchow	SSS-NAL	1	0.4
Human	S. enterica subsp. enterica (non-typed)	AMC-FOX-SSS	1	0.4
Human	S. enterica subsp. enterica (non-typed)	SSS	3	1.2
Human	S. enterica subsp. enterica (non-typed)	AMP-CHL-GEN-STR-TET-TGC	1	0.4
Human	S. enterica subsp. diarizonae	STR	1	0.4
Human	S. enterica subsp. salamae	SSS	1	0.4

Wild boar	S. enterica subsp. houtenae	SXT	1	0.4
Wild boar	S. enterica subsp. diarizonae	SXT	1	0.4
Wild boar	S. enterica subsp. houtenae	SXT	1	0.4
